# Supplementary material for: Modeling glioblastoma heterogeneity as a dynamic network of cell states
Source: Mol Syst Biol. 2021 Sep 16;17(9):e10105. doi: 10.15252/msb.202010105 (PMC8444284; doi:10.15252/msb.202010105)
Supplement: Supplementary file 6 — Source Data for Figure 5 [file MSB-17-e10105-s004.zip › Figure5A_sourcedata/GSEA_3071/hallmarks_stateVI.GseaPreranked.1623742557031/HALLMARK_MYOGENESIS.html]

Details for gene set HALLMARK\_MYOGENESIS[GSEA]

|  || Dataset | state63071 |
| Phenotype | NoPhenotypeAvailable |
| Upregulated in class | na\_neg |
| GeneSet | HALLMARK\_MYOGENESIS |
| Enrichment Score (ES) | -0.43116623 |
| Normalized Enrichment Score (NES) | -1.9594353 |
| Nominal p-value | 0.0011890606 |
| FDR q-value | 0.04259473 |
| FWER p-Value | 0.068 |
Table: GSEA Results Summary

  

Fig 1: Enrichment plot: HALLMARK\_MYOGENESIS      
 Profile of the Running ES Score & Positions of GeneSet Members on the Rank Ordered List

  

| PROBE | GENE SYMBOL | GENE\_TITLE | RANK IN GENE LIST | RANK METRIC SCORE | RUNNING ES | CORE ENRICHMENT || 1 | GADD45B |  |  | 90 | 0.758 | -0.0051 | No |
| 2 | TAGLN |  |  | 100 | 0.737 | 0.0119 | No |
| 3 | SPHK1 |  |  | 141 | 0.677 | 0.0186 | No |
| 4 | HBEGF |  |  | 172 | 0.627 | 0.0268 | No |
| 5 | KLF5 |  |  | 282 | 0.548 | 0.0109 | No |
| 6 | TEAD4 |  |  | 309 | 0.536 | 0.0178 | No |
| 7 | MYH9 |  |  | 312 | 0.534 | 0.0313 | No |
| 8 | COL6A2 |  |  | 335 | 0.521 | 0.0390 | No |
| 9 | PDLIM7 |  |  | 480 | 0.461 | 0.0110 | No |
| 10 | KIFC3 |  |  | 484 | 0.460 | 0.0223 | No |
| 11 | GPX3 |  |  | 493 | 0.457 | 0.0321 | No |
| 12 | SMTN |  |  | 498 | 0.455 | 0.0430 | No |
| 13 | TPM2 |  |  | 595 | 0.427 | 0.0275 | No |
| 14 | BIN1 |  |  | 836 | 0.371 | -0.0297 | No |
| 15 | TPM3 |  |  | 915 | 0.358 | -0.0420 | No |
| 16 | TNNT1 |  |  | 1041 | 0.338 | -0.0680 | No |
| 17 | LSP1 |  |  | 1048 | 0.337 | -0.0607 | No |
| 18 | MYO1C |  |  | 1059 | 0.336 | -0.0546 | No |
| 19 | IFRD1 |  |  | 1085 | 0.333 | -0.0528 | No |
| 20 | FLII |  |  | 1366 | 0.295 | -0.1232 | No |
| 21 | PDE4DIP |  |  | 1408 | 0.291 | -0.1269 | No |
| 22 | PSEN2 |  |  | 1442 | 0.286 | -0.1286 | No |
| 23 | FGF2 |  |  | 1557 | 0.274 | -0.1532 | No |
| 24 | ACSL1 |  |  | 1605 | 0.268 | -0.1592 | No |
| 25 | PRNP |  |  | 1866 | -0.260 | -0.2249 | No |
| 26 | EIF4A2 |  |  | 1904 | -0.264 | -0.2283 | No |
| 27 | SVIL |  |  | 1939 | -0.269 | -0.2306 | No |
| 28 | EFS |  |  | 1988 | -0.276 | -0.2367 | No |
| 29 | SORBS1 |  |  | 2277 | -0.329 | -0.3085 | No |
| 30 | KCNH2 |  |  | 2371 | -0.348 | -0.3252 | No |
| 31 | COL15A1 |  |  | 2379 | -0.349 | -0.3179 | No |
| 32 | AGL |  |  | 2450 | -0.367 | -0.3278 | No |
| 33 | MEF2A |  |  | 2638 | -0.409 | -0.3692 | No |
| 34 | MRAS |  |  | 2759 | -0.444 | -0.3909 | No |
| 35 | ATP6AP1 |  |  | 2779 | -0.449 | -0.3844 | No |
| 36 | GABARAPL2 |  |  | 2812 | -0.461 | -0.3811 | No |
| 37 | MYL6B |  |  | 2818 | -0.462 | -0.3703 | No |
| 38 | AEBP1 |  |  | 3037 | -0.557 | -0.4164 | Yes |
| 39 | GSN |  |  | 3041 | -0.559 | -0.4025 | Yes |
| 40 | ERBB3 |  |  | 3116 | -0.596 | -0.4074 | Yes |
| 41 | EPHB3 |  |  | 3117 | -0.596 | -0.3916 | Yes |
| 42 | NOTCH1 |  |  | 3192 | -0.657 | -0.3949 | Yes |
| 43 | RB1 |  |  | 3229 | -0.697 | -0.3865 | Yes |
| 44 | SPARC |  |  | 3356 | -0.822 | -0.4000 | Yes |
| 45 | CDH13 |  |  | 3410 | -0.913 | -0.3906 | Yes |
| 46 | IGFBP7 |  |  | 3428 | -0.958 | -0.3701 | Yes |
| 47 | COL3A1 |  |  | 3430 | -0.960 | -0.3450 | Yes |
| 48 | CHRNA1 |  |  | 3433 | -0.963 | -0.3201 | Yes |
| 49 | PKIA |  |  | 3492 | -1.090 | -0.3074 | Yes |
| 50 | NCAM1 |  |  | 3514 | -1.168 | -0.2824 | Yes |
| 51 | WWTR1 |  |  | 3519 | -1.185 | -0.2522 | Yes |
| 52 | APP |  |  | 3562 | -1.427 | -0.2262 | Yes |
| 53 | ITGA7 |  |  | 3572 | -1.466 | -0.1900 | Yes |
| 54 | CKB |  |  | 3579 | -1.575 | -0.1500 | Yes |
| 55 | IGFBP3 |  |  | 3588 | -1.623 | -0.1093 | Yes |
| 56 | CLU |  |  | 3601 | -1.801 | -0.0651 | Yes |
| 57 | CRYAB |  |  | 3633 | -2.841 | 0.0014 | Yes |
Table: GSEA details [plain text format]

  

Fig 2: HALLMARK\_MYOGENESIS: Random ES distribution      
 Gene set null distribution of ES for **HALLMARK\_MYOGENESIS**
